# Invited Commentary: Broadening the Evidence for Adolescent Sexual and Reproductive Health and Education in the United States

**DOI:** 10.1007/s10964-014-0178-8

**Published:** 2014-09-09

**Authors:** Amy T. Schalet, John S. Santelli, Stephen T. Russell, Carolyn T. Halpern, Sarah A. Miller, Sarah S. Pickering, Shoshana K. Goldberg, Jennifer M. Hoenig

**Affiliations:** 1 Department of Sociology, University of Massachusetts Amherst, Amherst, MA USA; 2 Heilbrunn Department of Population and Family Health, Columbia University, New York, NY USA; 3 Norton School of Family and Consumer Sciences, University of Arizona, Tucson, AZ USA; 4 Department of Maternal and Child Health, Gillings School of Global Public Health, University of North Carolina at Chapel Hill, Chapel Hill, NC USA

## Abstract

Scientific research has made major contributions to adolescent health by providing insights into factors that influence it and by defining ways to improve it. However, US adolescent sexual and reproductive health policies—particularly sexuality health education policies and programs—have not benefited from the full scope of scientific understanding. From 1998 to 2009, federal funding for sexuality education focused almost exclusively on ineffective and scientifically inaccurate abstinence-only-until-marriage (AOUM) programs. Since 2010, the largest source of federal funding for sexual health education has been the “tier 1” funding of the Office of Adolescent Health’s Teen Pregnancy Prevention Initiative. To be eligible for such funds, public and private entities must choose from a list of 35 programs that have been designated as “evidence-based” interventions (EBIs), determined based on their effectiveness at preventing teen pregnancies, reducing sexually transmitted infections, or reducing rates of sexual risk behaviors (i.e., sexual activity, contraceptive use, or number of partners). Although the transition from primarily AOUM to EBI is important progress, this definition of evidence is narrow and ignores factors known to play key roles in adolescent sexual and reproductive health. Important bodies of evidence are not treated as part of the essential evidence base, including research on lesbian, gay, bisexual, transgender, queer, and questioning (LGBTQ) youth; gender; and economic inequalities and health. These bodies of evidence underscore the need for sexual health education to approach adolescent sexuality holistically, to be inclusive of all youth, and to address and mitigate the impact of structural inequities. We provide recommendations to improve US sexual health education and to strengthen the translation of science into programs and policy.

## Introduction

Science is an essential foundation for adolescent sexual and reproductive health. Researchers, policy makers, advocates, and citizens accept science as a basis for policies and programs related to adolescent sexual and reproductive health. Scientific methods are used to identify the magnitude of adolescent health problems, contributing factors and health consequences, and to develop and evaluate health education and prevention programs. Scientific understanding of adolescent sexual and reproductive health encompasses general and discipline-specific scientific theory, qualitative and quantitative data, and scientific findings from diverse fields, including the medical, health, social, and behavioral sciences.

Adolescent sexual and reproductive health policy in the United States has failed to benefit from the full scope of science. From 1998 to 2009, federal funding for sexuality education focused almost exclusively on ineffective and scientifically inaccurate abstinence-only-until marriage (AOUM) programs (Santelli et al. 2006). President Obama’s 2010 teen pregnancy prevention initiative requires funded programs to be based in scientific evidence, but the implementation of this initiative has led to a new problem: “Evidence” is now narrowly defined to include only certain kinds of scientific findings. Currently, this initiative is the largest federal funding program addressing the sexual health needs of adolescents. The US Department of Health and Human Services has approved funding for 35 “evidence-based” programs selected on the basis of studies that have shown their effectiveness at preventing teen pregnancies, reducing sexually transmitted infections (STIs), or reducing rates of sexual risk behaviors (i.e., sexual activity, contraceptive use, or number of partners). These “evidence-based-interventions” (EBIs) are modeled after clinical trials and implemented with the intention to effect targeted behavior change.

While a clear advance over previous policy, current adolescent sexual and reproductive health policy and programming remain uninformed by the scientific base in profound ways. First, federal policy continues to fund abstinence programs that remain at odds with scientific thinking about adolescent sexual health. Second, key bodies of science are not treated as part of the essential evidence base for policy and programming. The exclusive focus on pregnancy and disease prevention in the definition of sexual health leaves out aspects of adolescent sexual development and health that researchers argue are critical, such as sexual orientation and gender beliefs. The focus on individual-level proximate causes of pregnancy and disease, such as sexual activity and contraceptive use, largely eclipses the systematic, society-level structural inequities that shape adolescent sexual behavior and risk. Finally, defining “evidence” as evaluations of program effectiveness for changing specific individual behaviors excludes broader evidence regarding psychological, cultural, and economic factors known to shape adolescent sexual health.

We begin our critique by reviewing the sexual and reproductive health needs of adolescents, with attention to the role of schools in promoting sexual health. We then discuss the emergence of evidence-based interventions as a guiding force in US adolescent sexual and reproductive health policies and programs. With this background, we examine three bodies of science—lesbian, gay, bisexual, transgender, queer and questioning (LGBTQ) youth and health; gender; and economic inequalities—identifying key findings that should inform adolescent sexual health and education programs. These bodies of evidence underscore the need for sexual health education to approach adolescent sexuality holistically, to be inclusive of all youth, and to address and mitigate the impact of structural inequities. In conclusion, we provide recommendations to improve adolescent sexuality education programs and policy, and the link between research and policy.

## Adolescents and Their Sexual and Reproductive Health

The World Health Organization definition of health is **“**a state of complete physical, mental, and social well-being and not merely the absence of disease or infirmity” (World Health Organization 1946). Definitions of reproductive health and sexual health mirror and expand upon this definition of health. Central to our conception of adolescent sexual and reproductive health is an understanding of adolescence as a life stage defined by physiological, psychological, social, and cultural transitions marking the movement from childhood to adulthood. Adolescents are emerging as adults, embodying a tension between the need for protection and guidance by parents and adult caregivers, on one hand, and the rights to autonomy and agency on the other.

Over the past 60 years, important changes have occurred in the timing of adolescent transitions, including age at first sex, length of educational preparation, age at marriage and timing of childbearing. In the United States, as in other developed countries, adolescents typically initiate sexual contact during their mid or late teens or early 20s (Finer 2007; Halpern and Haydon 2012). The establishment of constructive and satisfying romantic relationships is a key developmental task of adolescence and an important contributor to sexuality and sexual health (Mayer et al. 2008). We define sexuality to include the feelings, identities, relationships, and interactions that form the foundations of sexual development, and a variety of non-coital and coital sexual experiences. Important aspects of adolescent sexual development include maintaining a positive body image, developing self-efficacy in sexual decision-making and interactions, and forming mutually respectful romantic relationships (Schalet 2011a; Tolman 2002). Multiple and multi-level factors influence personal attitudes, motivations, and experiences, and can bolster or hinder the development of sexual self-efficacy, resiliency, healthy relationships, and positive body image, as well as behaviors that promote and protect or threaten health. Bodies of knowledge related to inter-personal dynamics, school climate, social norms, and cultural values and beliefs all provide information about the motives for adolescent sexual behavior (Russell 2005; Schalet 2011b). At the macro level, social and cultural forces including daily realities such as poverty or economic inequality, structural racism and stigmatization of youth who do not conform to rigid gender and sexual orientation norms, as well as medical technologies and access to health care and education, all profoundly shape adolescent health (Resnick et al. 2012). Social and behavioral science research on adolescent health has defined the important roles of families and peers, media, schools, life opportunities, demographic transitions, and cultural forces in shaping young people’s health status (Sawyer et al. 2012). Research in medicine and public health has documented the importance of health services, sexual health education, access to screening and treatment for STIs, and public policies in improving health.

### Adolescent Sexual Health Challenges

The need for more broad and effective translation of science into sexual and reproductive health policy is underscored by the significant sexual health burdens among youth. Comprising only 25 % of the sexually active population in the United States, young people (13–24 years) account for approximately half of the 20 million STIs contracted annually, including one in four of the estimated 50,000 new human immunodeficiency virus (HIV) infections diagnosed each year (CDC 2013a; Weinstock et al. 2004). Racial, ethnic, and gender disparities are striking. The majority of new adolescent cases (57 %) are among Blacks/African-Americans, with an additional 20 % occurring among Hispanics/Latinos (CDC 2012a, b). Women accounted for one in four new HIV cases in 2009; the incidence rate for Black/African American females (38.1/100,000) is 20 times the rate for White females (1.9) (CDC 2012b). However, men who have sex with men (MSM) are the population most affected by HIV in the United States; the estimated number of new HIV infections among adolescent and emerging adult Black/African American MSM (aged 13–29 years) increased 48 % from 2006 to 2009 (CDC 2011). Moreover, the US Centers for Disease Control and Prevention (CDC) reports that nearly 1.5 million high school students are affected by dating violence annually, with rates of violence and sexual coercion especially high among LGBTQ youth and female adolescents (CDC 2006). Finally, despite historic declines in adolescent pregnancy and teen births, US teen birth rates remain the highest among the developed nations (National Research Council 2013) even though levels of sexual experience are similar. Within the United States, poor, rural and minority women have higher teen birth rates. These disparities (by poverty and by race and ethnicity) demonstrate the critical need for effective and medically accurate sexuality education, accessible adolescent health care, and policy initiatives that reshape the educational and work opportunities for disadvantaged youth.

Despite the omnipresence of sexual messages in US media, frank public conversations about sexual and reproductive health, as holistically defined by organizations such as the World Health Organization, are rare. Discomfort with adolescent sexuality runs throughout the diverse institutions of American society, and it is perhaps no surprise that this discomfort has shaped our political conversations and policymaking. This discomfort and its impact on policy are not, however, inevitable. Other developed nations, such as the Netherlands and Denmark, have shifted away from a historical discomfort with adolescent sexuality, fostering national dialogue and policies aimed at supporting youth in their development—both sexual and socioeconomic—and seeing better overall adolescent sexual and reproductive health outcomes (Rose 2005; Singh et al. 2001; Schalet 2011b). In the United States, by contrast, multiple factors have contributed to ongoing controversy around adolescent sexuality, including its explicit politicization in recent decades, particularly with regard to the teaching of sexual health education within schools (Irvine 2004; Luker 2007).

### Adolescent Sexual Health Education

Schools have an essential role in promoting adolescent sexual and reproductive health, and science is essential in guiding the development of school health policies. As of fall 2013, about 50.1 million children and young people were enrolled in public elementary and secondary schools across the United States (National Center for Education Statistics 2013). Schools offer a practical and efficient means to reach young people with health information and health services. Because they include students across the socioeconomic spectrum, public schools can educate and serve children and youth who may not have access to education and services elsewhere. Schools are also an opportunity to educate all young people about health and sexuality before they initiate health risk behaviors, and to provide health services that prevent disease and promote health. Thus schools can help young people establish healthy behaviors that endure into adulthood (CDC 2013b; Kirby 2002). In addition to promoting healthy behaviors, schools are important venues for the development of norms and values and for fostering positive self-concept and agency around sex, sexuality, and relationships (Center for School, Health, and Education, 2011).

Educators, psychologists, and sociologists have argued, moreover, that sexual health education also has an important role to play in combating the health and social disparities that young people face. They note that structural racism, poverty, gender inequality, and the stigmatization of LGBTQ people all negatively impact health outcomes, including sexual health outcomes, and have argued that it is incumbent upon educators not to perpetuate inequalities within the classroom through explicit or implicit stereotyping (Fine and McClelland 2006). They point to problems not only in formal curricula, but also in the informal or hidden curricula—the implicit messages embedded in sexual health education—through which educators may inadvertently promote class, gender, and race stereotypes (Fields 2008; Morris 2007). Sexual health education has the potential to give young people the opportunity to critically examine the societal inequalities linked to gender, race, sexuality, and poverty (Fields 2008; Fine and McClelland 2006). Thus, while sexual health education cannot remove the inequalities in society at large, it can aid students in acquiring the critical thinking skills that will allow them to more effectively confront and challenge them.

## From “Ab-only” to “EBI-only”: US Federal Sexual Health Education Policy

The history of sexuality education in the United States reflects philosophical clashes and alternating domination of competing approaches stemming from those philosophical differences (Irvine 2004; Luker 2007; Moran 2002). Opponents of sexual health education have argued that teaching young people about sex encourages them to be sexually active, even though there is no evidence to support such claims; rather sexual health education helps some young people delay initiation of sex (Kirby et al. 2007a). In the 1990s the CDC provided significant funds to promote HIV/AIDS prevention education, which greatly expanded youth exposure to sexual health education but emphasized prevention of STIs and HIV. Beginning in 1998, federal funding shifted increasingly to a narrow focus on abstinence as the primary program and policy solution for “the problem” of adolescent sexuality. AOUM programs reflect the strong moral and religious beliefs of their authors. Key among those are the beliefs that sex outside of heterosexual marriage is sinful and that teaching about the health benefits of condoms and contraception is morally wrong because it encourages premarital sex. These beliefs are a critical feature of the “conceptual” basis for AOUM policies (Santelli et al. 2006).

From 1998 to 2009 the US government spent almost 2 billion dollars on assistance to states, community-based, and faith-based organizations for AOUM educational programs [Sexuality Information and Education Council of the United States (SIECUS 2014)]. Since 2009, US funding for domestic and international AOUM programs has decreased. However, federal and state-funded AOUM programs remain widespread in many parts of the United States, despite multiple scientific and human right concerns that have been raised by mainstream medical and public health organizations, including concerns about scientific accuracy, the withholding of life-saving information from young people, a lack of program efficacy, promotion of gender and racial stereotypes, insensitivity to non-heterosexual youth, and harm to traditional sexual health education (American Academy of Pediatrics Committee on Psychosocial Aspects of Child and Family Health and Committee on Adolescence 2001; American Civil Liberties Union 2008; American Public Health Association 2006; Santelli et al. 2006).

Rigorous evaluations of AOUM or abstinence-based curricula have failed to demonstrate efficacy in delaying initiation of sexual intercourse, reducing number of partners, increasing condom use, or promoting secondary abstinence (i.e., cessation of sexual intercourse among sexually experienced youth) (Kirby 2008; Trenholm et al. 2008). In contrast to abstinence approaches, a 2012 CDC meta-analysis of 66 comprehensive risk reduction programs for youth showed favorable effects on current sexual activity, frequency of sexual activity, number of sex partners, frequency of unprotected sexual activity, use of protection (either condoms and/or hormonal contraception), pregnancy, and STIs (Chin et al. 2012). In the same report, the CDC found insufficient scientific evidence for change in behaviors or other outcomes from abstinence education programs (Chin et al. 2012).

Since 2010, there has been a shift in federal approaches to sexual health education away from AOUM programs, and towards “evidence-based interventions” (EBI), led by the US federal Office of Adolescent Health (OAH) (AOUM programs have still received substantial funding through the Title V State Abstinence Education Grant Program). EBIs are treatments or interventions designed to effect behavior change that have been evaluated using randomized or quasi-experimental designs.^1^ US Federal health policy has increasingly relied on these EBIs (CDC 2013c; Evidence Based Intervention Network 2011). The authorizing language for the Office of Adolescent Health specifically describes “replication” of evidence-based approaches to teen pregnancy prevention programs and requires medical accuracy in all funded programs. In practice this means that to be eligible for the largest funding stream (termed “Tier 1”), grantees must select from and replicate with fidelity the now 35 programs that have been designated as EBIs.^2^ (A second group of funded programs, Tier 2, develops and tests new approaches to prevent teen pregnancy, including emphasis on underserved populations.)

Current emphasis on EBIs has been an important scientific and translational advance over prior federal efforts; however, a number of limitations have become evident with this approach. Current policy has focused on replication of specific curricula rather than the theory derived from research on EBIs, which provides a guide for understanding efficacy and adapting programs to new circumstances (Kirby et al. 2007b; UNESCO 2009). Further, the definitions of the health problems to be addressed and the types of evidence brought to bear on those problems omit central bodies of research. A narrow focus on disease and pregnancy prevention—and on the individual-level behavioral antecedents—undermines a more holistic approach to adolescent sexual health, and also ignores decades of scientific evidence of the ways that structural inequities shape adolescent sexual behavior and risk (Anderson et al. 2005). By defining the “evidence base” as evaluation research about program success in effecting specific (often small) behavior changes, the broader scientific record about factors known to shape adolescent sexual health outcomes has been relegated to a discretionary rather than central position. Grantees may use the breadth of scientific thinking to design tier two programs but they are not required—nor are those tasked with exact replication able—to integrate it into their programming. Thus, federal program requirements have had the unintended consequence of ignoring and marginalizing a broader body of scientific principles and evidence regarding adolescent sexual health and behavior.

## Toward Holistic Adolescent Sexual Health

We have argued that Evidence Based Interventions often do not reflect factors that the broad scientific literature identifies as key to health behaviors and risks, and do not approach individual behavior in the broad context of adolescents’ lives. As such, there is a disconnect between research and theoretical advances on one hand, and sexual health education programs and policies on the other (Romero et al. 2011). For instance, social and behavioral science research documents the significance of the sexual orientation of young people, the gender beliefs and inequities that shape their sexual agency and relationships, and the economic and racial inequalities that constrain their options, as crucial to a holistic understanding of adolescent sexual health. But many EBIs do not fully address or even acknowledge the psychosocial and structural factors that shape the ways in which adolescents conduct their sexual lives. Thus, while consensus has emerged across disciplines that gender, racism, stigmatization of LBGTQ youth, and poverty are critical to adolescent health, we lack programmatic emphasis and EBIs that address these inequalities. Moreover, when EBIs fail to address non-EBI scientific data about the role of poverty, race, and gender in adolescent sexual health they create the potential for reinforcing cultural stereotypes.

In the remainder of this article, we turn to evidence from across the social and behavioral sciences that should be central to all adolescent sexual health education. We show how the emerging research on LGBTQ youth calls for inclusiveness in adolescent sexual health education programming. Drawing from an extensive literature on the harmful effects of gender inequity and stereotypes, we demonstrate the need for sexual health education to address these issues. Finally, we illustrate how poverty and inequality intersect with adolescent sexual health education in a myriad of ways that have distinct implications for policy and programming.

### LGBTQ Education and Health

Contemporary LGBTQ and gender nonconforming youth “come out” or disclose their identities at younger ages than prior cohorts and have distinct sexual health needs (Floyd and Bakeman 2006). It is now commonly understood that LGBTQ students may face victimization at school, or generally hostile school climates (Birkett et al. 2009). Their needs are often invisible in sexual and reproductive health services, and they are typically excluded from sexual health education programs (Bay-Cheng 2003; Cianciotto and Cahill 2003; Sanchez 2012). Yet the known risks for LGBTQ youth are clear: greater rates of HIV for males and transgender youth; higher rates of high-risk sexual behavior for males, females, and transgender youth; and higher rates of pregnancy for both girls and boys (results for transgender youth are unknown) (Mustanski et al. 2011; Saewyc et al. 1999; Saewyc et al. 2009).

The focus of sexual health education historically has been on heterosexual sexuality, with emphasis on procreation, presumably or explicitly directed to the confines of marriage (Carter 2001). For more than 100 years, educators have grappled with the issue of how to teach youth about sexuality while promoting premarital chastity and marital monogamy, a dilemma that has often led to sacrifices of scientific accuracy in favor of ideology (Carter 2001). As a result LGBTQ youth are often excluded or left without relevant and necessary information to make safe and effective choices. Despite potential breadth, the dominant focus of sexuality education programs initially focused on the public health outcomes such as the prevention of unintended pregnancy, and since the mid-1980s, prevention of HIV/AIDS and STIs. Before HIV/AIDS, there was mostly silence on LGBTQ sexualities in sexual health education. Debates in the late 1990s became dominated by abstinence in sexual health education, a stark contrast to growing scientific knowledge about the efficacy of comprehensive sexuality education. In addition to other faults described above, the introduction of AOUM programs actively thwarted momentum to include LGBTQ youth needs in sexual health education by emphasizing abstinence until heterosexual marriage among high-school youth in different-sex relationships. Only since 2004 has marriage for same-sex couples been possible (to date more than a dozen states and the District of Columbia permit same-sex couples to marry); thus, for many LGBTQ youth, the AOUM message actively erases potential for comprehensive sexual health education. Moreover, some abstinence-only program content includes unequivocally hostile messages about LGBTQ people (Cianciotto and Cahill 2003).

Several empirical studies have begun to document the ways that abstinence programs may undermine LGBTQ youth sexual health and well-being (Kosciw et al. 2012). One report showed that compared to schools with other types of sexuality education, LGBTQ students who attended schools that taught abstinence-only programs faced greater harassment in the form of anti-LGBTQ remarks. Further, by excluding sexual minorities (or in some cases giving disparaging information about them), abstinence-only programs may produce feelings of rejection and being disconnected to school (Kosciw et al. 2012). These feelings may lead to negative mental health outcomes such as depression and anxiety and serve as precursors for other health risk behaviors (Almeida et al. 2009; Kosciw et al. 2012). On the other hand, there is evidence that inclusive strategies can promote sexual health for LGBTQ students. For example, Blake et al. (2001) found that LGB students in schools with gay-sensitive HIV instruction reported lower sexual risk taking and substance use.

Not only may LGBTQ students be invisible or marginalized in sexuality education, but their health needs may not align with the sexual health education needs of students involved in different-sex relationships or sexual activity. If the risk for disease is presented only with reference to penile-vaginal sexual behaviors, there may be deleterious consequences for the health of those who engage in same-sex relationships or sexual activity. For example, HPV poses a threat to all male and female youth, including cancer risk stemming from same- as well as different-sex sexual activity. However, if education only refers to heterosexual vaginal transmission, youth may erroneously conclude that HPV risk pertains only to heterosexual vaginal sex. Such an approach would obscure other sexual behaviors that pose risk for HPV, such as non-penetrative sexual contact, even though the prevalence of HPV among women who have never engaged in vaginal intercourse is high, as is the risk for anal cancer associated with HPV among men who engage in receptive anal intercourse (Mayer et al. 2008). Heterosexual bias in sexuality education will leave some youth without critical knowledge they need to make safe sexual choices.

Only nine states require that sexual health education programs provide inclusive information on sexual orientation (Guttmacher Institute 2013). Seven states (and multiple localities) have laws that expressly forbid discussion of LGBTQ issues (including sexual health and HIV/AIDS awareness) in a positive light, if at all; of those, three states (Alabama, South Carolina, and Texas) require that sexuality education programs include negative messages about same-sex sexuality (Guttmacher Institute 2013; McGovern 2012). Alabama law criminalizes same-sex relationships and sexual behavior and proclaims them to be “not a lifestyle acceptable to the general public.” Additionally, the law asserts that this position comes from a “factual manner and from a public health perspective.” This discriminating law is not unique; there are other laws throughout the country that work to stigmatize LGBTQ people, including youth in the classroom, by expressly forbidding discussion of LGBTQ issues in a positive manner (McGovern 2012). Meanwhile, two proposed federal laws have languished; the *Safe Schools Improvement Act* (2013 S. 403) and the *Student Non*-*Discrimination Act* (2013 HR 1652) would explicitly provide protection to LGBTQ students in US schools, and create a supportive policy context for inclusive health policies and programs.

In spite of this discouraging context for sexuality education, the pace of social change regarding LGBTQ inclusion has been extraordinary, as evidenced, for example, by the growing number of US states and other nations that permit marriage for same-sex couples. Beyond sexuality education programs, there is an emerging body of evidence that documents specific educational practices and strategies that create positive school climates for LGBTQ youth, including inclusive anti-discrimination and anti-bullying policies and laws, school personnel training and advocacy, access to LGBTQ-related resources and curricula, and gay-straight alliance (GSA) school clubs (Russell et al. 2010). A number of studies show that these strategies are linked to adolescent academic achievement and mental and behavioral health (Blake et al. 2001; Goodenow et al. 2006; Poteat et al. 2013).Thus, a growing body of evidence points to principles for promoting adolescent health in ways that respect and include LGBTQ youth, and that respond to known inequities many LGBTQ youth experience. These principles should inform the evidence base for federal sexual health education programs and policies.

### Gender and Sexual Health Education

A second area in which current scientific thinking and sexual health education policy and programs are not aligned concerns the impact of gender (in)equity and gender norms. Research across disciplines has demonstrated that gender norms and inequities are key factors in shaping health generally, and sexual health in particular (Rogow and Haberland 2005). International health organizations have recognized that promoting gender equity is critical to advancing health across the life course (World Health Organization 2002). Domestically, Healthy People 2020 includes gender and gender identity as dimensions linked to health disparities—that is, systematic obstacles to health—and it aspires to reduce those disparities. But in the “Adolescent Health” section, the document is silent about the need to address gender inequities or harmful gender beliefs.^3^ Establishing gender equity and challenging gender beliefs that research has shown to be harmful to adolescent sexual health have never been central goals in US adolescent sexual health and education policy (DeLamater 2007). In fact, many abstinence-only and abstinence-only-until-marriage programs have taught gender stereotypes as facts (Curran 2011; Delamater 2007; Fine and McClelland 2006). Even approaches that include information beyond abstinence have perpetuated gender inequities through gender stereotyping implicit in curricula or teachers’ informal communications (Curran 2011; Fields 2008; Garcia 2009, 2012).

It has long been established among researchers that gender inequities, and the gender ideologies that uphold them, are key factors in shaping sexual and reproductive health globally and domestically, affecting STIs, HIV/AIDS, unintended pregnancies, and sexual violence (Rogow and Haberland 2005; Santana et al. 2006). Scholars have documented how traditional gender roles impede women’s sexual autonomy and self-efficacy, and thereby increase their vulnerability to STIs and HIV, intimate partner violence, unwanted sex, and unintended pregnancy (Amaro and Raj 2000; Amaro et al. 2001; Impett et al. 2006; Jewkes 2010; Phillips 2000). Gender-based relational power imbalances impact women’s capacity to advocate for their own sexual safety (Phillips 2000; Rosenthal and Levy 2010). For instance, compared to women who report low levels of relationship power, women with higher levels are five times as likely to report consistent condom use (Pulerwitz et al. 2002). Cultural beliefs about gender can also have negative health consequences for men by, for instance, encouraging risk behavior (Higgins et al. 2010).

Gender ideologies shape how youth view and experience themselves and each other. Researchers have documented how schools, peer culture and other institutions overtly and covertly communicate distinct gender ideologies about sex and romance to young people (Chambers et al. 2004; Eder et al. 1995; Fields 2008; Pascoe 2007). Traditional gender ideologies frequently link masculinity with heterosexual sexual activity, sex drive, sexual initiation, and lack of emotional involvement, and femininity with sexual passivity, sexual restraint, responsibility for controlling boys’ desires, and emotional over-involvement (Allen 2003; Bay-Cheng 2003). The sexual double standard, which encourages and celebrates heterosexual sexual experience in teenage boys but censures and stigmatizes sexual experience in teenage girls, is endemic in the United States, though it varies by local context and culture (Crawford and Popp 2003; Greene and Faulkner 2005; Marston and King 2006).

The sexual double standard harms girls by stigmatizing their sexual desires and experiences, reducing their negotiating power within sexual encounters, and conditioning girls to believe that their own desires and wishes are less significant than those of their male partners (Hamilton and Armstrong 2009; Holland et al. 1998; Martin 1996; Tolman 2002). Negative cultural beliefs about girls’ sexuality can make it difficult for them to disclose their sexual histories to partners, parents, or adult care providers (Greene and Faulkner 2005; Schalet 2011a, b). Traditional gender roles can also hinder girls in refusing unwanted sex and insisting on condom use (Impett et al. 2006; Kirkman et al. 1998; Petitifor 2012). Possessing a sense of sexual self-efficacy—a sense that one has power over one’s sexual decision making—seems to be especially important in aiding girls to engage in safer sex behaviors (Gutierrez et al. 2000; Pearson 2006). There is additional evidence to suggest that when girls know about, and feel entitled to, sexual pleasure, they are better able to advocate for themselves and their sexual health, leading scholars to call on sexual health education to challenge the double standard and emphasize the value of girls’ desires and pleasure (Hirst 2013; Horne and Zimmerbeck 2006; Impett et al. 2006; Martin 1996; Tolman 2002).

Boys are also disadvantaged by prevailing gender ideologies. The sexual double standard can make it appear as if boys should always desire sex, and never say no to sex, even risky sex (Bowleg et al. 2000). The prevailing ideologies stigmatize boys’ emotional vulnerabilities and needs, including their needs for intimate friendships and romantic relationships, making them less prepared to have intimate relationships (Giordano et al. 2006; Way et al. 2013). They also stigmatize homosexuality and behaviors associated with homosexuality (Kimmel 2008; Klein 2012; Pascoe 2007). Norms about appropriate male behavior affect all males. But those who adhere most to “traditional” beliefs about masculinity—for instance, that men should be tough, have status in society, not behave in ways marked as “feminine,” and regularly have heterosexual sex— are most at risk for negative consequences compared to other boys and men. Those who embrace such traditional attitudes toward masculinity tend to also report more sexual partners, engage in more unprotected vaginal sex, and show less self-efficacy and consistency in condom use (Noar and Morokoff 2002; Pleck et al. 1993, 1994; Santana et al. 2006; Shearer et al. 2005).

There is growing evidence that among adult men some masculine gender norms are linked to violence in intimate relationships (Gallagher and Parrott 2011; Murnen et al. 2002). For example, compared to other men, men who report more traditional masculinity ideologies are more likely to report having perpetrated violence or sexual coercion (Marín et al. 1997; Santana et al. 2006). Conversely, compared to less egalitarian men, men whose gender role ideologies are more egalitarian report fewer instances of physical aggression against their intimate partners (Fitzpatrick et al. 2004). Gender norms also shape young people’s capacities to resist, report, and recover from sexual violation. Boys are unlikely to report sexual coercion due to homophobia as well as masculinity norms that emphasize male sexual desire and strength and obfuscate boys’ capacity to be coerced or intimately violated (Bullock and Beckson 2011). For girls, the pressure to be normatively feminine (sexually passive, accommodating, “nice”) can make resistance to unwanted sexual advances difficult (Armstrong et al. 2006; Hamilton and Armstrong 2009; Phillips 2000). The stigma around girls’ sexuality also prevents many from seeking help, a barrier that is heightened for low-income girls and girls of color (Collins 2005; Froyum 2010).

In short, there is strong and consistent evidence that gender beliefs and (in)equities shape sexual health (Rogow and Haberland 2005). However, until recently, these areas have received very little attention in US adolescent sexual health policy and programming (Grose et al. 2014; Rolleri 2013a; Rolleri 2013b). There is no requirement for federally-funded sexuality education to work toward gender equity, avoid explicit or implicit gender stereotyping, or include modules that help students challenge harmful gender beliefs. Abstinence-oriented programs have often taught gender stereotypes as fact (DeLamater 2007; Fine and McClelland 2006; Curran 2011).^4^ Approaches that include information beyond abstinence can also perpetuate gender ideologies through the topics they cover and leave out, or include implicit gender stereotyping in apparently gender-neutral exercises and role plays (Bay-Cheng 2003; Curran 2011; Fields 2008). Unless harmful gender beliefs are explicitly addressed and challenged, sexual health education runs the risk of reinforcing those beliefs through the taken-for-granted assumptions teachers and students bring into the classroom (Fields 2008; Garcia 2009, 2012; Froyum 2010). Yet, of the 35 designated (Tier 1) “evidence-based” programs, only a handful (all of which target youth of color) even mention gender in their program description, suggesting incorrectly that only minority groups contend with harmful gender beliefs (Office of Adolescent Health 2014a).^5^ The research record shows the advisability of ensuring that all sexual health programs are free from harmful gender beliefs—which may be explicit or implicit in the curricula—and include tools to help students address and challenge these beliefs.

### Poverty, Inequality, and Sexual Health Education

Considerable literature has demonstrated that poverty and economic inequalities are fundamental barriers to positive youth development. Youth subject to these inequalities have lower academic achievement, and are more likely to leave school early, thereby compounding cumulative socioeconomic effects on health. Youth in poverty are also more likely to engage in delinquent behavior, to become sexually active early, and to have elevated risk of STIs, unintended pregnancies, and non-marital births (Brooks-Gunn et al. 1997; Dinkelman et al. 2008; Duncan and Rodgers 1988; Duncan et al. 2010; Grantham-McGregor et al. 2007). Youth in poverty also lack access to quality health services (National Research Council 2009). The effects of persistent poverty are especially pernicious, affecting socio-emotional development and health, and increasing the likelihood of enduring ill effects into adulthood.

The deleterious effects of poverty are critical considerations for adolescent health and development in the United States, where low-income students now comprise a near majority of public school children in the United States.^6^ About one in six of all youth and one in three African American youth ages 12–17 live in families with incomes below the official poverty level.^7^ In addition to the close linkage between minority racial/ethnic status and poverty, there are major racial disparities in long-term exposure to neighborhood poverty. Analyses of data from the Panel Study of Income Dynamics indicate that 40 % of African Americans experience sustained exposure to high-poverty neighborhoods, versus 5 % of non-Blacks (Wodtke 2013).

The negative consequences of poverty are a function of the structural and experiential inequalities that typify the life contexts of impoverished youth. Poor youth are more likely to live in neighborhoods characterized by adverse physical and social environments, with higher rates of crime and limited access to recreational facilities and after-school programs, and are more likely to attend lower quality schools with fewer resources (Murry et al. 2011). They are also less likely to have access to mental and physical health services. Exposure to poverty during adolescence may be especially important, given adolescents’ expanding social world. Recent analyses suggest that sustained exposure to neighborhood poverty substantially increases the risk of becoming an adolescent parent, and that exposure during adolescence may have a greater effect than exposure earlier in childhood (Wodtke 2013). Further, poverty shapes sexual network structure, increasing the likelihood of STIs (Fichtenberg et al. 2010). These contexts mold adolescents’ sexual knowledge, perceptions about and access to contraception, and their hope for the future.

Poverty intersects with individual and structural characteristics to generate significant health disparities, the cumulative health differences that result from obstacles linked to factors such as race/ethnicity, gender, disability, sexual orientation, and gender identity. A recent review of health disparities in the United States (CDC 2013d) documented persistent race/ethnicity disparities in health outcomes, access to health care, adoption of health promoting behavior, and exposure to health promoting environments, with no evidence of a temporal decrease between 2005 and 2009. Documented disparities, beyond those related to sexual and reproductive health, include differences in chronic conditions such as asthma, diabetes and hypertension, as well as differences in mortality from causes such as coronary heart disease, stroke, drugs, homicide, suicide, and vehicle related injuries (CDC 2013d).

Thus, the adverse impact of poverty is compounded by racism, sexism, heterosexism, and discrimination against individuals with disabilities. These prejudicial belief systems reflect irrational biases toward members of a certain race, biological sex, sexual orientation, gender identity, or level of ability on the basis that a certain group is “superior/inferior” or “normal/abnormal.” Structural racism/sexism/heterosexism, that is, “macrolevel systems, social forces, institutions, ideologies, and processes that interact with one another to generate and reinforce inequities among… groups” (Gee and Ford 2011; p. 116), normalizes and legitimizes unequal treatment and discrimination Structural discrimination can take many forms, including social segregation (e.g., neighborhood, schools, health care facilities) and exclusionary immigration policy, and can persist across generations through the cumulative effects of interacting systems. For example, because of racial discrimination in the real estate industry African Americans are considerably more likely to live in poor neighborhoods, even if economic resources would permit residing in non-poor neighborhoods (Iceland and Scopilliti 2008).

The Intersectionality Framework (see for example, Weber and Parra-Medina 2003) proposes that characteristics such as race, class and gender are not distinct social categories. They reflect multidimensional and overlapping experiences that are a function of mutually reinforcing social processes and institutions. The intersection of microlevel identities and macrolevel structural factors can affect health by producing and sustaining economic inequality via groups’ access to social, economic, and political resources and privileges. The effects of neighborhood disadvantage on school dropout, for example, are twice as large for African American youth versus their White peers (Crowder and South 2003). Community poverty levels also contribute to LGBT youth’s experiences in school; youth in higher poverty communities report more victimization in school because of sexual orientation and gender expression than those in more affluent communities (Kosciw et al. 2009). Poverty and racial segregation can also affect the sexual expectations and behavior of youth, leading youth in these contexts to consider early sexual activity as normal and even expected. Youth in low income neighborhoods may not have access to educational and occupational opportunities, and may view sexual activity as a pathway to social status rather than an obstacle to socioeconomic achievement (Ramirez-Valles et al. 2002).

The intersections of poverty, inequality, structural discrimination, and adolescent sexual and reproductive health are numerous. Sexual health education exists within a variety of structural and social contexts (Fine and McClelland 2006). Sexuality affects, and is affected by, complex interactions between individual biopsychosocial factors and a host of economic, political and cultural factors. Sexuality and sexual rights are thus interwoven with broader human rights and the sociopolitical issues that affect those rights, such as economic inequality and structural racism. Approaching adolescent sexual health with an eye toward poverty and its intersections with diverse social identities means attention to not only material deprivation but also to social and political exclusion and restrictions on rights, including sexual rights (Armas 2007), that are linked to behavior. Poverty limits knowledge about and access to sexual and reproductive health services, constrains positive sexual expression and feelings of self-efficacy, and makes disadvantaged youth vulnerable to sexual exploitation and violence. This is why the experience of poverty is associated with greater sexual risk-taking (e.g., early sexual onset, multiple sexual partnerships, lack of condom use) in both the United States and global contexts (Brooks-Gunn et al. 1997; Dodoo et al. 2007; Duncan and Rodgers 1988).

US policy makers must understand and address the importance of poverty’s complex intersections with diverse identities and the impact on how youth respond to sexuality education. Sexual health education paradigms and curricula often assume adolescents are in school and that they live in homogeneous social and physical environments free of economic or other social barriers. Sexuality education may explicitly or inadvertently reinforce cultural stereotypes about young people of color, who are more likely to be poor, as sexually irresponsible (Fine and McClelland 2006; Fields 2008; Garcia 2009, 2012). Similarly, sexual health education may presume “proper” relationships and family forms that are less common among low income youth or youth of color. With few economic opportunities and resources to develop positive sexual identities, low income or minority youth may rely on rigid, exclusionary, and ultimately counterproductive frameworks to assert self- and group-worth (Froyum 2007). Failure to recognize erroneous assumptions and the lived reality of youth can lead to unintended effects on adolescent sexuality, promoting exclusion of teens who do not conform to expected gender and sexual norms and ultimately failing to reduce inequality (Bedford 2008; Drucker 2009). Sexual health education must thus recognize the diverse life course trajectories and family formations that characterize students’ lives. In addition, scholars have argued, sexual health education must create opportunities for students to discuss sexual agency and risks in the context of their broader life aspirations and the multifold factors that constraints those aspirations (Fields 2008; Fine and McClelland 2006; Rogow and Haberland 2005). Although sexual health education cannot remove the structural disparities, by giving young people the opportunity to critically examine the inequalities they encounter, it can bolster their ability to respond to them.

Policy makers must also promote adolescent sexual and reproductive health by investing in youth through multi-faceted and multi-level poverty alleviation efforts that build youth assets and promote health. Despite frequently voiced concerns about the intractable nature of poverty (and by extension, hopelessness), the United States has a track record of intentional and effective large-scale implementation of poverty alleviation. In the late 1950s 22 % of US residents lived in poverty; after the launch of the War on Poverty in the 1960s, that percentage had dropped to 11 % by 1973 (Council of Economic Advisors 2014). Changes were even more drastic among the elderly, who once had the country’s highest poverty rates, but whose chances of living in poverty have been sharply reduced through programs such as Social Security and Medicare (Fischer et al. 1996).

Today, the poverty rate of US children and teens is among the highest in the industrial world. Given the pervasive detrimental effects on youth development, poverty alleviation programs are vital to improving adolescent sexual and reproductive health. Indeed, comparing across five developed nations, where rates of sexual activity among youth were similar, Singh and colleagues report a strong association between the higher US teen birth rate and the greater proportion of teens who grow up poor (Singh et al. 2001). Yet there is strong evidence that structural interventions can both directly and indirectly improve adolescent health, and that large-scale implementation is both feasible and successful (Snell et al. 2013). In many European, Latin American, and African countries, governments offer a variety of income supplements, especially to families with children. Singh et al. (2001) point toward policies that are likely to affect adolescent sexual and reproductive health specifically, including national health care systems and government investment in job training and opportunities for young people, easing the transition into adulthood, facilitating long-term planning, and reducing the motivation to have a child prematurely. The authors conclude “improving adolescents’ socioeconomic status is a way to prevent their having poor reproductive health outcomes—not only unplanned or early pregnancies or births, but also STDs” (p. 258).

Policy makers should heed lessons learned from our country’s success in reducing poverty among the elderly, and from other countries’ successes in better promoting adolescent sexual and reproductive health by investing in multi-faceted and multi-level poverty alleviation efforts that build youth assets and promote health. For individual adolescents, efforts are needed to enhance adolescents’ motivation for personal and professional achievement (e.g., healthy interpersonal relationships, education and occupation), and avoid behaviors that increase risks of STIs, poor emotional and physical health, and early pregnancy and childbearing. We also need to target structural barriers created by economic, racial and ethnic inequalities (e.g., increase resources for high-poverty schools), and offer support services to help families and their children (e.g., adequate funding for Title X family planning clinics) as they move toward better financial security, without predication of assistance on particular family structures that may not be feasible for or desired by all individuals.

## Conclusions and Recommendations

US federal sexual health policy has come a long way since the introduction of AOUM policies when federally funded programs were often medically inaccurate, were prohibited from teaching the health benefits of condoms and contraception, and were required to teach students that sex outside of heterosexual marriage would damage them. In providing our critique we acknowledge the strides that have been made in current federal policies and initiatives, and we also acknowledge that US sexual health education programs and policies exist in a cultural and political context that is not fully conducive to holistic approaches to adolescent sexual health education, or to the full range of contemporary science in this field. The current “evidence-based” policy, while a significant leap forward, is limited in a number of ways. The US federal policy continues to fund abstinence-only programs as part of its Teen Pregnancy Prevention Initiative as well as other funding streams. But more important, the definition of scientific evidence is limited to a narrow understanding of what constitutes the broad scientific evidence for adolescent sexual and reproductive health. The current policy does not require programs to be engaged with the breadth of current scientific thinking about adolescents and their sexual health.

We have sought to highlight the limitations of EBIs by examining three bodies of literature on topics about which there is growing scientific consensus. This evidence indicates that adolescent sexual health is undermined by the exclusion and stigmatization of LGBTQ youth, gender inequities and stereotypes, and poverty and structural racism. Likewise, the research shows that greater inclusiveness, more gender and economic equity, and freedom from harmful stereotypes, all benefit young people and their sexual health. And yet, although there are some excellent programs that approach adolescent sexuality holistically (see for instance, International Sexuality and HIV Curriculum Working Group 2011), federal policy does not require its recipients of funds to address these critical topics, and indeed very few federally funded programs do.^8^ When federally funded sexual health education does not intentionally address these topics, it may overtly or inadvertently promulgate gender, sexual orientation, class, and racial stereotyping, and fail to give youth resources to combat them. Gender, heterosexual, economic and racial biases in sexual health education leave youth without the personal agency and the critical knowledge they need to make safer sexual choices.

Based on these considerations, we offer several recommendations for federal sexual health education policy, as well as for more effective translation of science into policymaking and programming. First, adolescent sexual and reproductive health policy should be based on scientific input from a broad range of disciplines, including social, behavioral, medical, and public health sciences. The full range of scientific evidence should guide adolescent sexual and reproductive health policies, including adolescent sexual health education. Federally-funded programs must address gender, poverty, and lesbian, gay, bisexual, transgender, queer, and questioning (LGBTQ) youth. Federal policy makers should engage in conversation with the broad range of scientific communities and professional societies. Policy makers and federal program administrators must draw on scientific advisors to help translate the broader evidence base, and guide the development of interventions that reflect current scientific thinking. Further, scientists must become actively engaged in the translation of their work for policy and practice.

Second, sexual health education should be inclusive of a wide range of viewpoints and populations without stigmatizing any group. It should avoid heteronormative approaches and aim to strengthen young people’s capacity to challenge harmful stereotypes. In cooperation with scientists and health professional associations, content guidelines should be established for federally-funded sexuality education programs to assure medical accuracy as well as gender equity and inclusion of LGBTQ youth. This should be a priority across federal agencies and throughout the Department of Health and Human Services, including the CDC, Administration for Children and Families, and the Office of Adolescent Health (in particular, in its next round of teen pregnancy prevention programs).

Finally, sexuality education programs and policies must acknowledge the role that structural and contextual factors play in sexual risk. Comprehensive sexuality education should recognize personal, interpersonal, social, economic and cultural factors that shape adolescents’ sexual motivations and behaviors. A fundamental goal must be the removal of economic, gender and LGBTQ disparities in adolescent sexual and reproductive health through laws, regulations, and funding requirements.

Structural inequalities that are critical barriers to adolescent sexual health promotion are at the heart of some of the most contested issues in American society: the sexual orientation of adolescents, concepts of gender, and economic and racial inequalities. When federally funded health interventions do not engage directly with these issues, and thus ignore the broader scientific consensus regarding adolescent sexual and reproductive health, they run the risk of reproducing these inequalities (Fine and McClelland 2006). By incorporating the full range of scientific evidence regarding adolescent sexual and reproductive health, federal, state, and local efforts will be best positioned to promote adolescent health and well-being.

## Footnotes

1 Such research designs are particularly useful in evaluating behavioral interventions where effect sizes are small or where the efficacy of an intervention is unknown.

2 The Personal Responsibility Education Program (PREP) of the US Office of the Administration for Children and Families is a distinct funding stream for teen pregnancy prevention; it does not require use of an EBI, however grantees are highly encouraged to use programs on the EBI list.

3 One of the goals in this section is to increase the proportion of secondary schools that “prohibit harassment based on a student’s sexual orientation or gender identity.” This goal is important but does not in itself encourage schools to promote gender equity or address the effects of harmful gender beliefs in sexual health education.

4 One recent study of popular abstinence-only program content found a softening of some older gender stereotypes alongside the emergence of new ones, including the manipulative female leading a male astray (Lamb et al. 2013).

5 Without a systematic content analysis of these programs, it is difficult to assess whether they merely mention gender or actively seek to change unhealthy gender norms. One tier 1, evidence-based intervention that does the latter is SiHLE (Rolleri 2013a). Encouragingly, under its Tier 2 funding, the Office of Adolescent Health has funded *Gender Matters*, an “innovative gender transformative program,” noting that “addressing gender norms is essential to improving the health of teens, but often prevention programs leave out these discussions” (Office of Adolescent Health 2014b, p. 1).

6 Defined by eligibility for the federal free and reduced-price meals program in the 2010–2011 school year; Suitts et al. 2013.

7 More than one in three of all young people, 12–17, live in “near-poverty” (often considered a more accurate measure).

8 Encouragingly, the federal government has, under its tier 2 funding for experimental programs, supported program development and evaluation in some of research areas we have highlighted, including a program to address and change harmful gender beliefs.
